# First-principles studies of substituent effects on squaraine dyes

**DOI:** 10.1039/d1ra01377g

**Published:** 2021-05-26

**Authors:** German Barcenas, Austin Biaggne, Olga A. Mass, Christopher K. Wilson, Olena M. Obukhova, Olga S. Kolosova, Anatoliy L. Tatarets, Ewald Terpetschnig, Ryan D. Pensack, Jeunghoon Lee, William B. Knowlton, Bernard Yurke, Lan Li

**Affiliations:** Micron School of Materials Science and Engineering, Boise State University Boise ID 83725 USA lanli@boisestate.edu; SSI “Institute for Single Crystals” of National Academy of Sciences of Ukraine Kharkov 61072 Ukraine; SETA BioMedicals Urbana IL 61802 USA; Department of Electrical and Computer Engineering, Boise State University Boise ID 83725 USA; Department of Chemistry and Biochemistry, Boise State University Boise ID 83725 USA; Center for Advanced Energy Studies Idaho Falls ID 83401 USA

## Abstract

Dye molecules that absorb light in the visible region are key components in many applications, including organic photovoltaics, biological fluorescent labeling, super-resolution microscopy, and energy transport. One family of dyes, known as squaraines, has received considerable attention recently due to their favorable electronic and photophysical properties. In addition, these dyes have a strong propensity for aggregation, which results in emergent materials properties, such as exciton delocalization. This will be of benefit in charge separation and energy transport along with fundamental studies in quantum information. Given the high structural tunability of squaraine dyes, it is possible that exciton delocalization could be tailored by modifying the substituents attached to the π-conjugated network. To date, limited theoretical studies have explored the role of substituent effects on the electronic and photophysical properties of squaraines in the context of DNA-templated dye aggregates and resultant excitonic behavior. We used *ab initio* theoretical methods to determine the effects of substituents on the electronic and photophysical properties for a series of nine different squaraine dyes. Solvation free energy was also investigated as an insight into changes in hydrophobic behavior from substituents. The role of molecular symmetry on these properties was also explored *via* conformation and substitution. We found that substituent effects are correlated with the empirical Hammett constant, which demonstrates their electron donating or electron withdrawing strength. Electron withdrawing groups were found to impact solvation free energy, transition dipole moment, static dipole difference, and absorbance more than electron donating groups. All substituents showed a redshift in absorption for the squaraine dye. In addition, solvation free energy increases with Hammett constant. This work represents a first step toward establishing design rules for dyes with desired properties for excitonic applications.

## Abstract 2

Squaraine dyes are candidates for DNA-templated excitonic interactions. This work presents substituent effects on the electronic and photophysicalproperties of squaraine dyes and a correlation between empirical Hammettconstant and those properties.

## Graphical abstract

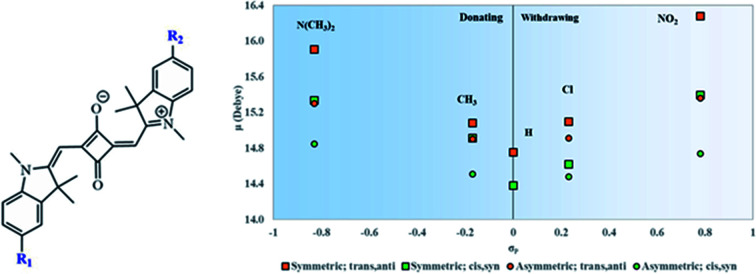

## 1. Introduction

The aggregation of dyes gives rise to Frenkel exciton delocalization in molecular composite systems.^1,2^ The study of dye aggregates can provide insight into the implementation of aggregate systems, such as organic photovoltaics,^3,4^ near infrared medical imaging^5^ and molecular photoswitch applications^6^ that draw from a well-established theoretical framework exploiting unique exciton transfer properties.^7^ Dye aggregate behavior is well described by the molecular exciton model, formulated by Davydov and Kasha, in which intermolecular dipole–dipole interactions lead to the mixing of excited-state wavefunctions to access nondegenerate states.^2,8,9^ When dye monomers aggregate, the monomeric excited-state energy levels split into the excited states of the aggregate, in which excitons are distributed in a wave-like fashion, called exciton delocalization.^10–12^ The new excited-state behavior (*i.e.*, exciton delocalization) in the dye aggregate generally manifests as an energy shift.^13,14^ The inclusion of a double-body exciton interaction introduces a second order molecular excitation interaction, *i.e.*, the exciton–exciton interaction energy *K*_*m*,*n*_,which is crucial for modeling exciton–exciton interaction behavior in the dye aggregate.^14–16^

It is pertinent to also discuss the environment, in which dyes may aggregate. DNA is an attractive choice for dye templating due to its customization at the nanoscale and an ability to promote the exciton delocalization of dyes.^17–34^ DNA has been shown to negligibly change the electronic properties of visible-light absorbing dye monomers.^35^ As such, the electronic properties of the dye monomers can be evaluated as free-dyes to screen their potential utility as DNA-templated dyes. The customizability of DNA templates and the options for binding dyes to different sites are further enhanced *via* DNA origami methods to construct multidimensional scaffolds.^31,36^

Expanding the number of dyes that can potentially be incorporated will no doubt increase the functional capabilities of DNA templating.^27,29,35,37–48^ Squaraine (SQ) dyes, a family of dyes similar to the widely used cyanine dyes, but with a central squaraine ring, have advantageous properties, such as strong absorption in the visible spectrum^49^ and resistance to photobleaching.^50^ They can potentially be structurally tailored for a wide variety of applications.^51–53^ First synthesized by Treibs and Jacobs,^54^ the central feature of SQ dyes includes an electron-deficient squaric moiety, combined with electron-rich groups in a symmetric manner by means of a methine bridge.^52^ The photophysical features and extensive structural tunability make squaraines well-suited candidates for the investigation of exciton delocalization when assembled.^18,24,28,55^ Although the dipole–dipole coulombic coupling between dyes must be considered to accurately predict aggregate absorption spectra, monomer transition dipole moments provide estimates of the strength of exciton delocalization for various dye configurations *via* the extended dipole approximation, which can be used as a guide for exciton applications.^15,56^

There is also a robust body of work demonstrating the customizability of squaraine dyes, offering opportunities for the tunability of dye properties through the engineering of functional groups to yield desired properties.^50,52,57–59^ Previous work on photophysical property engineering of squaraine dyes *via* substitution is present in the literature with an emphasis on the changing of the donor groups that flank the central squaraine group or the finetuning of dyes largely for efficient light-to-electrical conversion and imaging.^52,60,61^ In the context of promoting stronger exitonic interaction, customizing squaraine dyes enables the introduction of substituents that may alter their electronic structures to make them more favorable for dipole interactions without detrimentally affecting photophysical properties.^62–64^ There has also been a research interest in manipulating two key excitonic coupling factors, including exciton hopping (or exchange) energy, *J*_*m*,*n*_, and exciton–exciton interaction energy, *K*_*m*,*n*_, by controlling the transition dipole *μ* and static dipole difference Δ*d* of a dye monomer.^56,65–67^ The maximization of *μ* within a single absorption band is also benefitted by the minimization of vibronic coupling of dyes.^68^ Maximizing exciton–exciton interaction energy depends upon the maximization of Δ*d*. This should be concomitant with maintaining or increasing *μ* and is a primary target for the selection of dyes and their substituents in this study. The maximization of these quantities increases *J*_*m*,*n*_ and *K*_*m*,*n*_, leading to a larger excitation energy.^56^

Substituent effects may also alter the propensity for dye aggregation by changing dye solubility.^35,39^ Local environment impacts the orientation of the dye by introducing steric effects when hydrophobic substituents are added. In the case of DNA-templated squaraines, the local environment can consider both a solvent environment as well as DNA. The balance of substituents' ability to influence electronic, photophysical, and hydrophobic behaviors is key to the promotion of ideal dyes for excitonic device performance. To further investigate the effect of substituents, this study focuses on substituents that can increase the hydrophobicity of a squaraine dye, because this is expected to promote dye packing in order to influence dipole interactions.^39^ The study of substituent effects on the electronic, photophysical, and solubility properties of monomers can therefore provide information on candidates for aggregation.

In this work, first-principles methods were used to address the potential for indolenine-based squaraine dyes to be tuned for excitonic applications. Specifically, we used density functional theory (DFT)-based methods to calculate the ground- and excited-state properties of nine squaraine dyes, *i.e.*, SQ-H_2_, SQ-N(CH_3_)_2_, SQ-(N(CH_3_)_2_)_2_, SQ-CH_3_, SQ-(CH_3_)_2_, SQ-Cl, SQ-(Cl)_2_, SQ-NO_2_, and SQ-(NO_2_)_2_. A range of substituents on a free squaraine dye were studied to evaluate their influence on *μ*, Δ*d*, absorbance, and hydrophobicity, which were subsequently validated against experimental data for DNA-templated squaraine monomers. By adding functional groups to an unsubstituted squaraine, *i.e.*, SQ-H_2_, its *μ*, Δ*d*, absorbance, and hydrophobicity could be altered. In addition, three different conformers were investigated for each dye to examine the impact of structural changes.

## 2. Methodology

### 2.1 Computational methods

**Fig. 1:**
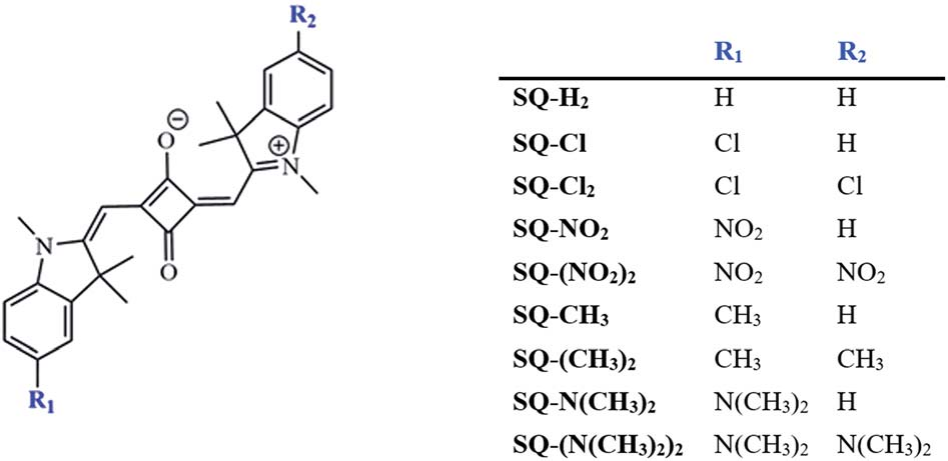
Indolenine-based squaraines. H atoms are located at the R_1_ and R_2_ positions, forming SQ-H_2_. If H is replaced with substituents, it forms either symmetric (R_1_ = R_2_ = substituent) or asymmetric (R_1_ = H and R_2_ = substituent). Substituents this study focuses on include N(CH_3_)_2_, CH_3_, Cl, and NO_2_.

The Gaussian 16 software package^69^ was used to perform density functional theory (DFT) and time-dependent density functional theory (TD-DFT) calculations. DFT has proven to provide insight in the investigation of dye properties,^65–67,70^ and there has been extensive work addressing best practices in employing this method, such as appropriate exchange-correlation functionals to represent electron–electron interactions in the fluorescing dyes similar to squaraine dyes.^71–74^ The dyes were built and initially relaxed with the molecular editing software Avogadro^75^ using the UFF^76^ method. All calculations were performed using the 6-31+G(d,p) basis set with the M06-2X^77^ exchange-correlation functional, because this showed a good agreement with experimental results in comparison with sets of similar fluorescent dyes.^72,73^ M06-2X is a hybrid meta-generalized gradient approximation exchange-correlation functional. This nonlocality denotes an inclusion of Hartree–Fock (HF) exchange energy, which is advantageous for non-metal systems. The term “meta” indicates that the functional is constrained to be optimized using empirical data. Specifically, for dipole and absorption calculations, M06-2X was found to be the most reliable in comparison with other popular pure and hybrid exchange-correlation functionals.^72,74,78^ Jacquemin *et al.* also conducted an extensive survey of exchange-correlation functionals applicable to different dyes, including squaraine and so-called push–pull dyes.^71,73,78–80^ Molecules were built according to the structures in Fig. 1. Comparisons were made to monomers incorporated to DNA. DNA was found to negligibly affect the absorbance data of a monomer.^18,35^

Atomic structures were optimized using a tight root mean square residual force of 1 × (10)^−5^ Hartree/Bohr and an ultra-fine integration grid of 99 radial shells and 590 angular points per shell. The ground-state optimization of these molecules was verified by ground-state frequency calculations to ensure that no imaginary frequencies were present, because imaginary frequencies represent unstable geometry. Dyes exhibiting desirable Δ*d* (*i.e.*, large Δ*d*) were selected for further calculations to determine vibrationally-resolved absorption spectra. To do so, the optimized first excited-state geometry was used to calculate the excited-state frequency to ensure that an optimized structure was achieved. This procedure resulted in an adiabatic transition by including the zero-point vibrational energies, which accounted for vibrational energies at the states' respective minima.^80^ The ground- and excited-state frequencies were then used to calculate an absorption spectrum for each molecule with the Franck–Condon (FC) approximation. The FC approximation assumes that nuclear motion is frozen on the timescale of the electronic transition. Our previous studies showed that the calculated absorption spectra of cyanine dyes with the FC approximation agreed well with experiments.^35^ Here, the changes in the bond lengths of ground and excited states of squaraine dyes were analyzed. We found that the bond lengths among the squaric moiety carbons elongated. The methine chain lengths, connecting the trimethylindolenine groups, and the carbon–oxygen bonds of the squaric moiety shortened. However, all the changes between ground and excited states were on the order of 0.01 Å. This finding indicated that the FC approximation was adequate for squaraine dyes, and agreed well with Bassal *et al.*^74^ The vertical excitation was expected to be indicative of the excitation behavior of the squaraine dyes. For dyes in solution, nonequilibrium solvent–solute conditions were considered. Permanent dipole information was generated by taking the vertical excitation from the optimized ground state. This vertical excitation could result in a difference between ground- and excited-state static dipoles, *i.e.* Δ*d*, written as:^72^1
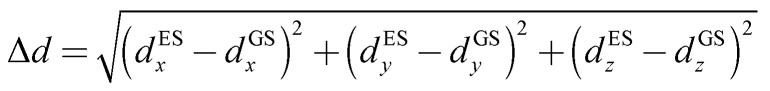
where d^j^_i_ is the Cartesian component of the permanent dipole moment, *i*, in either the excited ES or ground GS state, *j*.

Solvation energy, Δ*G*_solv_, has been shown to correspond to hydrophobic behavior and qualitatively imply the stability of dye aggregate.^35^ To investigate the likelihood of dye aggregation, the solvation free energy Δ*G*_solv_ was calculated by taking a difference in the ground state energies calculated using SMD (*i.e*., Solvation Model based on Density) water and vacuum given as:^35,81^

2 Δ*G*_solv_ = *E*_solv_ − *E*_v_

where *E*_solv_ is the ground-state energy calculated in solvent and *E*_v_ is the vacuum or gas-phase ground-state energy. Previous studies have introduced squaraine conformers present in solution. Their population percentages were calculated using the Boltzmann distribution.^58^ The energies used to compare likely populations were the ground-state energies of each system in vacuum and in a water environment at 25 °C. In addition to water solvent, pyridine, quinoline, and isoquinoline were also investigated to approximate a DNA environment. In order to reduce computational time, nitrogen heterocycle solvents were used as suitable analogs to nitrogenous purine and pyrimidine nucleobases in a DNA scaffold.

### 2.2 Experimental methods

**Fig. 2:**
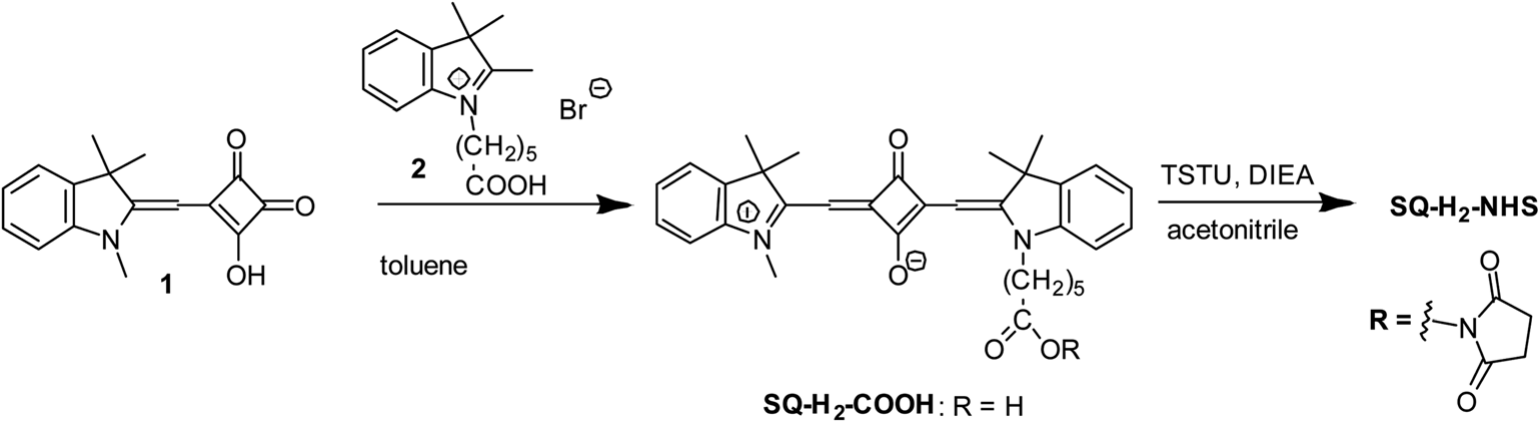
Synthesis of SQ-H_2_.

*N*-Hydroxysuccinimide ester of 2-((1-(5-carboxypentyl)-3,3-dimethylindolin-2-ylidene)methyl)-3-oxo-4-((1,3,3-trimethyl-3*H*-indol-1-ium-2-yl)methylene)cyclobut-1-en-1-olate (SQ-H_2_-NHS) was synthesised similar to the procedure described in Kolosova *et al.*^58^ (Fig. 2).

3-Hydroxy-4-((1,3,3-trimethylindolin-2-ylidene)methyl)cyclobut-3-ene-1,2-dione (1) (150 mg, 0.56 mmol) and 1-(5-carboxypentyl)-2,3,3-trimethyl-3*H*-indolium bromide (2) (200 mg, 0.56 mmol) were heated under reflux in toluene (10 mL) for 10 h. The solvent was removed under reduced pressure by a rotary evaporator. The residue was purified by a column chromatography (Silica gel 60, 0–8% methanol–chloroform) to give SQ-H_2_-COOH (190 mg, 65%) as a dark blue solid with a golden sheen. ^1^H-NMR (200 MHz, DMSO-d_6_), *δ*, ppm: 7.52 (2H, d, 7.3 Hz, arom.), 7.44–7.25 (4H, m, arom.), 7.25–7.04 (2H, m, arom.), 5.79 (1H, s, CH), 5.76 (1H, s, CH), 4.06 (2H, t, 7.4 Hz, NCH_2_), 3.57 (3H, s, NCH_3_), 2.21 (2H, t, 6.7 Hz, CH̲_2_COOH), 1.68 (12H, s, (CH_3_)_2_), 1.80–1.29 (6H, m). MALDI-TOF MS, *m*/*z* calcd. for [C_33_H_36_N_2_O_4_] 524.27, found: 525.32 [M + H]^+^. Anal. calcd. (%) for C_33_H_36_N_2_O_4_: C, 75.55; H, 6.92; N, 5.34. Found C, 75.43; H, 6.89; N, 5.31. UV-Vis: *λ*_max_ (Abs) 630 nm (methanol); *λ*_max_ (Em) 639 nm (methanol); *λ*_max_ (Abs) 622 nm, *ε* 285 000 M^−1^ cm^−1^ (phosphate buffer); *λ*_max_ (Em) 632 nm (phosphate buffer).

SQ-H_2_-COOH (30 mg, 57 mmol), *N*,*N*,*N*′,*N*′-tetramethyl-*O*-(*N*-succinimidyl)uronium tetrafluoroborate (TSTU) (26 mg, 86 μmol), and *N*,*N*-diisopropylethylamine (DIEA) (16 mL, 92 mmol) were dissolved in acetonitrile (3 mL). The solution was stirred at room temperature for 20 min. The solvent was removed under reduced pressure by a rotary evaporator. The residue was purified by a column chromatography (Silica gel 60, 0–3% methanol–chloroform) to give SQ-H_2_-NHS. Yield: 18 mg (51%). ^1^H-NMR (200 MHz, DMSO-d_6_), *δ*, ppm: 7.53 (2H, d, 7.4 Hz, arom.), 7.46–7.24 (4H, m, arom.), 7.24–7.01 (2H, m, arom.), 5.79 (1H, s, CH), 5.76 (1H, s, CH), 4.06 (2H, t, 7.4 Hz, NCH_2_), 3.58 (3H, s, NCH_3_), 2.81 (4H, s, CH_2_ (NHS)), 2.67 (2H, t, 6.0 Hz, CH̲_2_COONHS), 1.68 (12H, s, (CH_3_)_2_), 1.80–1.29 (6H, m).

For computation-experiment validation, we assembled a four-arm DNA Holliday junction with the unsubstituted squaraine SQ-H_2_ covalently attached to one of the oligonucleotides. Three unlabeled oligonucleotides and one oligonucleotide labeled with SQ-H_2_ (SETA BioMedicals, Urbana-Champaign, IL) *via* the nucleosidic sequence modifier C6 dT were obtained from Integrated DNA Technologies (Coralville, IA). Squaraine-labeled and unlabeled DNA oligonucleotides were rehydrated in ultrapure water (Barnstead Nanopure, Thermo Scientific) to prepare a 100 μM stock solution. Concentrations of DNA samples were determined spectroscopically on NanoDrop One Microvolume UV-Vis (Thermo Scientific) using a calculated extinction coefficient. DNA Holliday junctions were prepared by combining equimolar amounts of complimentary functionalized and non-functionalized oligonucleotides in 1× TBE 15 mM MgCl_2_ buffer solution, to a final DNA concentration 1.5 μM. Samples were annealed in a Mastercycler Nexus PCR cycler (Eppendorf) according to the following protocol: 4 min at 94 °C, followed by a cooling rate: 0.1 °C per 15 s from 94 °C to 64 °C, and 10 °C per minute from 64 °C to room temperature. UV-Vis spectra were recorded in duplicates at room temperature on a dual-beam Cary 5000 UV-Vis-NIR spectrophotometer (Agilent Technologies) in a cuvette with a 10 mm pathlength. Absorbance spectra were monitored over a wavelength range of 230–800 nm. Spectra were normalized at dye absorption maximum in UV-Vis range using OriginPro 2019.

## 3 Results

### 3.1 Boltzmann populations of conformers

**Fig. 3:**
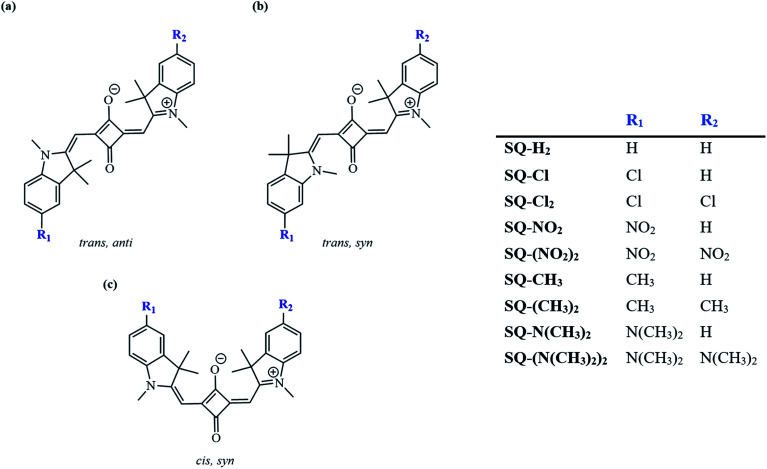
Three possible indolenine squaraine conformers: (a) *trans*,*anti*, (b) *trans*,*syn*, and (c) *cis*,*syn*. Substituents this study focuses on include N(CH_3_)_2_, CH_3_, Cl, and NO_2_.

To further complement changes associated with composition *via* substitution, this study considered three conformers of the indolenine-based squaraine, including *trans*,*syn* with *C*_s_ symmetry; *cis*,*syn* with *C*_2v_ symmetry; and *trans*,*anti* with *C*_2h_ symmetry using a Boltzmann distribution calculation at room temperature.^58^ The conformations of dyes were considered based on previously reported indolenine-based squaraines *via* photoisomerization.^58,82^ The energies of the optimized ground-state squaraine dyes were used to calculate the energy differences and corresponding Boltzmann populations of three conformers of the indolenine-based squaraine dyes, as shown in Fig. 3. The energy differences between the optimized ground-state conformers are shown in Table 1. For all dyes, a more stable conformer is represented by a more negative total energy. Comparing the differences of total energies of different conformers shows that the *trans*,*anti* conformer is about 5 kJ mol^−1^ more stable than the *cis*,*syn* conformer (*trans*,*anti*–*cis*,*syn*) and about 8 kJ mol^−1^ more stable than the *trans*,*syn* conformer (*trans*,*anti*–*trans*,*syn*). The energy difference results, when used as states for a Boltzmann distribution, demonstrate that the two major conformers of indolenine-squaraines are *trans*,*anti* and *cis*,*syn.* The *trans*,*anti* conformer is the most energetically favorable for all the indolenine-based squaraine dyes with the most negative total energy. These energy differences have been described as a result of steric strain from the dimethyl group of the indolenine rings in agreement with previous computational and experimental studies.^58,83,84^ The *trans*,*anti* conformer further benefits from a staggered conjugation, favoring minimized steric effects.^85,86^ However, we found that substituents have a minor effect on the Boltzmann populations with respect to the unsubstituted *trans*,*anti* squaraine (SQ-H_2_) at most by only 3%. The greatest variation of conformer population from the unsubstituted *cis*,*syn*SQ-H_2_ conformer occurs from SQ-Cl by 3%. The Boltzmann populations of different *trans*,*syn* conformers are lower than 4% and have the greatest variation with respect to the unsubstituted *trans*,*syn*SQ-H_2_ is also from SQ-Cl by 1%. Computational results show that the *cis*,*syn* and *trans*,*anti* conformers are popular, so our further studies focus on these conformers.

**Table 1:** Ground-state total energy differences between *trans*,*anti* and *cis*,*syn* conformers and between *trans*,*anti* and *trans*,*syn* conformers, as well as associated Boltzmann populations calculated from the energy differences at 25 °C in vacuum

Dye	Energy difference (kJ mol^−1^)		Boltzmann populations (%)		
	*trans*,*anti*–*cis*,*syn*	*trans*,*anti*–*trans*,*syn*	*trans*,*anti*	*trans*,*syn*	*cis*,*syn*
SQ-(N(CH_3_)_2_)_2_	−4.81	−8.39	84.93	2.88	12.2
SQ-N(CH_3_)_2_	−4.78	−8.18	84.58	3.12	12.3
SQ-(CH_3_)_2_	−4.77	−8.33	82.14	1.31	16.54
SQ-CH_3_	−4.76	−8.32	84.65	2.95	12.39
SQ-H_2_	−4.81	−8.31	82.69	2.57	14.74
SQ-Cl	−4.70	−8.18	80.49	1.49	18.02
SQ-(Cl)_2_	−4.72	−8.46	83.02	2.54	14.44
SQ-NO_2_	−4.69	−8.85	84.84	2.38	12.78
SQ-(NO_2_)_2_	−4.95	−8.73	85.83	2.53	11.63

### 3.2 Comparison of SQ-H_2_ with experiment

**Fig. 4:**
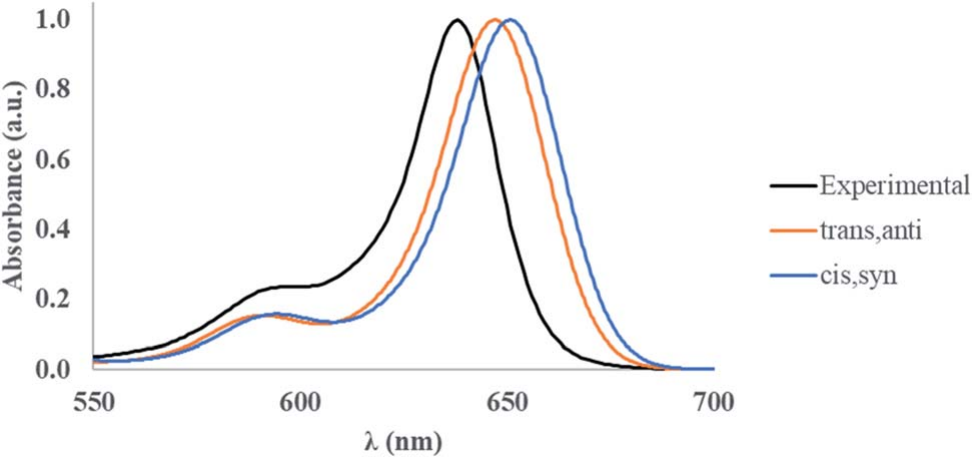
Experimental and calculated vibrationally-resolved absorption spectra for SQ-H_2_. The calculated absorption spectra were obtained using the FC approach with the optimized ground- and excited-state structures of a free dye in a water solvent. The experimental spectrum was obtained for SQ-H_2_ covalently attached to a DNA HJ; the concentration of SQ-H_2_ – DNA HJ construct was 1.5 μM in 1× TBE 15 mM MgCl_2_ aqueous buffer solution.

To validate our approach for the calculations of ground- and excited-state properties of substituted squaraines, the vibrationally-resolved absorption spectra of SQ-H_2_ were generated using the Franck–Condon (FC) method in the TD-DFT framework for the *trans*,*anti* and *cis*,*syn* conformers. The calculated absorption spectra are shown in Fig. 4 along with the experimental absorption profile.

TD-DFT satisfactorily reproduces the lineshape of the experimental absorption spectrum, which exhibits a strong absorption peak at 638 nm and a smaller vibrionic shoulder around 590 nm. The TD-DFT calculated peak absorption *λ*_max_ is found to be 647 nm for the *trans*,*anti* conformer and 651 nm for the *cis*,*syn* conformer. Compared to the experimental data, the absorbance data calculated by TD-DFT exhibits a peak absorption *λ*_max_ within 0.027 eV of experiment (as calculated for the *trans*,*anti* conformer). Furthermore, the calculated *trans*,*anti* peak absorption *λ* is closest to experiment, which suggests that the *trans*,*anti* conformer dominates the dye populations, agreeing well with the Boltzmann population results (Table 1). The deviation from the theoretical calculation is a known artifact in TD-DFT calculations when computing absorption spectra for dyes. This is due to small perturbations in the excited electronic density that the hybrid exchange-correlation functional cannot adequately model in the TD-DFT scheme.^87^

### 3.3 Solvation free energy calculations

**Fig. 5:**
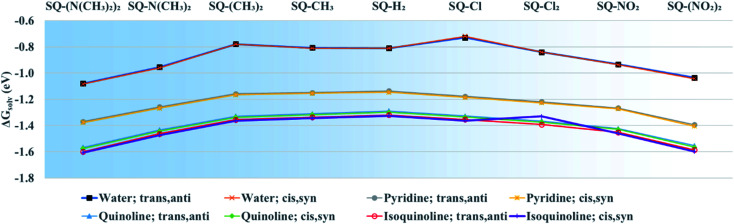
Solvation free energy (Δ*G*_solv_) of unsubstituted and symmetrically substituted squaraine dyes in water, pyridine, quinoline, and isoquinoline calculated using eqn (2). Geometry optimizations were done using the M06-2X functional. The lines added to the data are to highlight trends of the data and are not meant to infer quantitative behavior. The *x*-axis is in order of increasing donating and withdrawing strength as its position away from SQ-H_2_.

To determine the effects of electron donating and withdrawing substituents on the solvation energies of squaraine dyes, DFT ground-state optimization calculations were performed in vacuum and solvent. From the solvated and vacuum energies, the solvation free energy, Δ*G*_solv_, was determined using eqn (2) for each dye, as shown in Fig. 5. As with other studies,^88,89^ the values of Δ*G*_solv_ were calculated to estimate the solubility of the dyes in the given solvent. As shown in Fig. 1, the substituted dye structures consist of symmetrically substituted dyes (R_1_ = R_2_) and asymmetrically substituted dyes (R_1_ = H ≠ R_2_).

Unsubstituted squaraine has a Δ*G*_solv_ of −0.81 eV in water for both the *trans*,*anti* and *cis*,*syn* conformers. For all dyes, the conformer does not affect Δ*G*_solv_, and, overall, the values of Δ*G*_solv_ are only slightly affected by substitution. Upon substitution, most dyes exhibit more negative Δ*G*_solv_ values in water, except for SQ-Cl, which has the least negative Δ*G*_solv_ value, indicating being the most hydrophobic. In contrast, SQ-(N(CH_3_)_2_)_2_ has the most negative Δ*G*_solv_ for the water solvated dyes and so is taken to be the most hydrophilic. The Δ*G*_solv_ for the dyes in pyridine, quinoline, and isoquinoline follow similar trends as those in water. The values of Δ*G*_solv_ in pyridine, quinoline, and isoquinoline are also unaffected by conformers. Δ*G*_solv_ for the dyes in water are less negative, indicating that it is more energetically favorable for the dyes to form solutes in pyridine, quinoline, and isoquinoline. These three solvents are taken to mimic the molecular structure of DNA bases.

### 3.4 Excited-state calculations

The optimized water-solvated ground-state structures of unsubstituted and substituted squaraines were used for single-step TD-DFT calculations in the first excited singlet state to determine the effects that electron donating and electron withdrawing substituents have on electronic excited-state properties (*i.e.*, *μ* and Δ*d*). We introduced the empirically derived Hammett constant (*σ*_p_), which could quantify the strength of a substituent as electron withdrawing (positive) or electron donating (negative).^90,91^ A list of these constants is provided in Table 2. N(CH_3_)_2_ and NO_2_ are strong donating and withdrawing substituents, respectively. CH_3_ and Cl are weak donating and withdrawing substituents, respectively. The Hammett constant has been shown to relate to the characteristics of an electronic structure.^67,92^ To establish a relationship between the strengths of the electron donating and electron withdrawing substituents (as quantified with *σ*_p_) and calculated properties, *μ*, Δ*d*, and Δ*λ*_max_ are plotted against the *σ*_p_ of attached substituents, as discussed in the following sections.

**Table 2:** Substituents used in this study and their corresponding Hammett constants,^90,91^*σ*_p_. The empirically derived Hammett constant (*σ*_p_) is used to quantify the strength in which a substituent is electron withdrawing (positive) or electron donating (negative)

Substituent	Hammett constant (*σ*_p_)	Classification
N(CH_3_)_2_	−0.83	Donating
CH_3_	−0.17	
H	0	
Cl	0.23	Withdrawing
NO_2_	0.78	

#### 3.4.1 Transition dipole moments

**Fig. 6:**
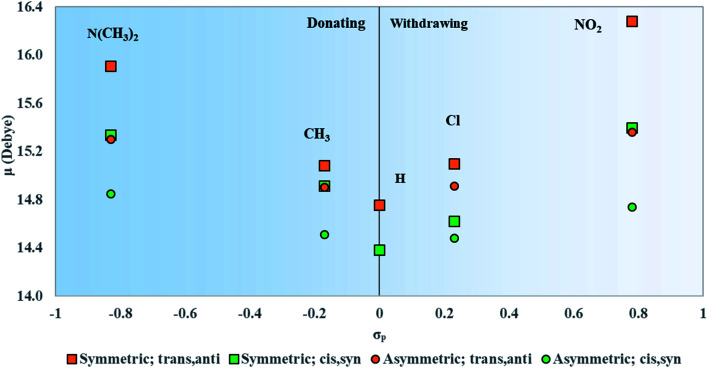
Transition dipole moment magnitudes (*μ*) for symmetrically and asymmetrically substituted squaraines plotted against the Hammett constant of the substituent(s) attached to the dye.

Compared to unsubstituted squaraine, Fig. 6 shows that substituents with the larger magnitudes of *σ*_p_ yield dyes with larger *μ*. The calculated values of *μ* for unsubstituted squaraine (SQ-H_2_) are 14.7 D for the *trans*,*anti* conformer and 14.4 D for the *cis*,*syn* conformer and were determined to be along the long axis of the dye. The dyes exhibiting the largest values of *μ* are *trans*,*anti*SQ-(N(CH_3_)_2_)_2_ (15.9 D) and SQ-(NO_2_)_2_ (16.3 D), which are the dyes with the strongest electron donating and electron withdrawing substituents, respectively. In general, symmetrically substituted dyes have the larger values of *μ* than asymmetrically substituted ones. Furthermore, the *trans*,*anti* conformers exhibit the larger values of *μ* than *cis*,*syn* conformers. In comparison with the unsubstituted squaraine, the largest change is SQ-(NO_2_)_2_ with 1.6 D, and all substituents contribute an increase in *μ*.

#### 3.4.2 Static dipole differences

**Fig. 7:**
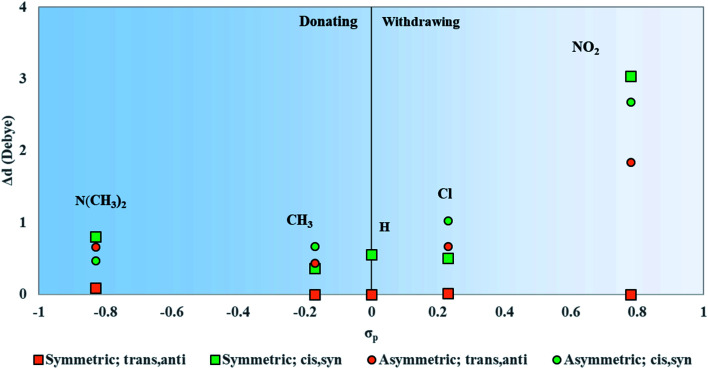
Static dipole difference magnitudes (Δ*d*) for symmetrically and asymmetrically substituted squaraines plotted against the Hammett constant of the substituent(s) attached to the dye.

Similar to *μ*, the Δ*d* of squaraine increases with the magnitude of *σ*_p_, as shown in Fig. 7. Substitution type and conformation also influence Δ*d*. Symmetrically substituted SQ-N(CH_3_)_2_ and SQ-NO_2_ dyes have the higher Δ*d* than their asymmetrical ones. Symmetrically substituted SQ-(CH_3_) and SQ-Cl dyes have the lower Δ*d* than their asymmetrical ones. Furthermore, symmetrically substituted dyes in the *trans*,*anti* conformations all have Δ*d* values of nearly 0 D while others have non-zero Δ*d*. In general, t*rans*,*anti* conformers, characterized by *C*_2h_ type symmetry, have the lower values of Δ*d* compared to *cis*,*syn* conformers, characterized by *C*_2v_ symmetry. The largest values of Δ*d* belong to *cis*,*syn*SQ-Cl (0.8 D) in the asymmetrically substituted dyes and *cis*,*syn*SQ-NO_2_ (3.0 D) in the symmetrically substituted dyes. This indicates that electron withdrawing substituents have the larger impact on Δ*d* than electron donating substituents.

#### 3.4.3 Absorption spectra of asymmetric squaraines

**Fig. 8:**
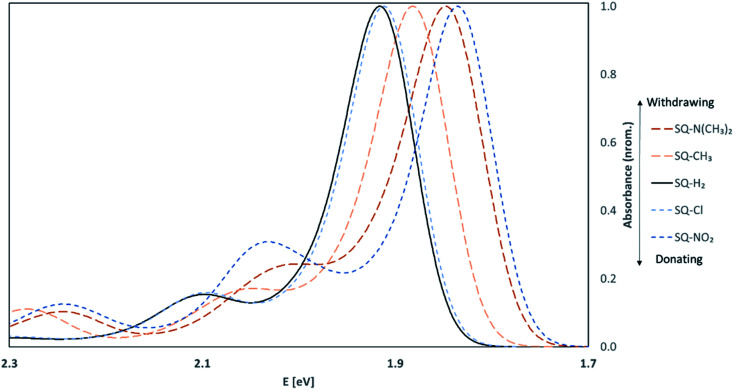
Vibrationally-resolved absorption spectra for *trans*,*anti* asymmetrically substituted squaraine dyes calculated using the FC method in implicit water. The solid black line is the unsubstituted squaraine (SQ-H_2_) absorbance spectrum.

To further test the effects of substituents on the excited-state properties of dyes, vibrationally-resolved absorption spectra were calculated. The asymmetrically substituted dyes in the *trans*,*anti* conformations with the larger Δ*d* values were chosen for further studies with the FC method. The normalized results are shown in Fig. 8.

Upon asymmetric substitution, the lineshapes of the spectra remain relatively unaffected, with a main absorption peak between 1.97 and 1.84 eV (630 and 675 nm) and a smaller vibrionic shoulder around 2.1 eV (600 nm). However, the *λ*_max_ values for asymmetrically substituted dyes are redshifted compared to the unsubstituted squaraine dye. SQ-Cl has the smallest redshift of 0.04 eV. The largest redshifts of 0.08 eV and 0.09 eV belong to SQ-N(CH_3_)_2_ and SQ-NO_2_, indicating that the stronger electron donating or electron withdrawing substituents have a larger effect on *λ*_max_.

**Fig. 9:**
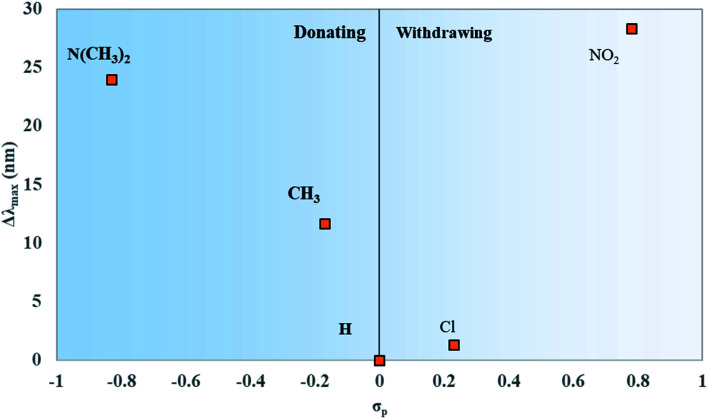
Maximum absorption wavelength differences from unsubstituted squaraine (Δ*λ*_max_) for asymmetrically substituted squaraines plotted against the Hammett constant of the substituent(s) attached to the dye.

Like *μ* and Δ*d*, the values for Δ*λ*_max_ were plotted against the values of *σ*_p_ for the substituents attached to the dyes. Δ*λ*_max_ is defined as the redshift of the *λ*_max_ of the dyes in Fig. 8 from unsubstituted squaraine (SQ-H_2_). As shown in Fig. 9, the larger *σ*_p_ values of the substituents promote the larger Δ*λ*_max_.

## 4. Discussion

In regard to the changes to Δ*d* and *μ* upon substitution, all substituents lead to changes in electronic structures as compared with the reference squaraine SQ-H_2_. In the frame of treating the squaraine as a donor–acceptor–donor dye, the substituents modify the donating behavior of the trimethylindolenine groups attached to the accepting squaric moiety. The electron withdrawing substituents in this study appear to have a stronger effect on the change of Δ*d* and *μ*. Electronically, this may be the result of decreasing the donating ability of the indolenine groups towards the squaraine center. In contrast to the withdrawing substituents, the donating substituents are expected to increase the donating strength of the indolenine groups. The contribution of the electron donating substituents to an increase in Δ*d* and *μ* is relatively smaller than that of the electron withdrawing substituents (Fig. 6 and 7).

Δ*G*_solv_ is sensitive to solvent and becomes more negative with the nitrogen heterocyclic solvents pyridine, quinoline, and isoquinoline, as shown in Fig. 5. This trend increases from pyridine to quinoline and isoquinoline and can be attributed to π–π interactions between the solvent and the solute.^93^ The magnitude of Δ*G*_solv_ also increases with *σ*_p_. An increase in the magnitude of Δ*G*_solv_ indicates a greater stability in a solvent environment. This would suggest that the dyes have a propensity for a DNA environment and are more likely to aggregate in a DNA template rather than dissolve in aqueous solution.

Recent squaraine dye applications have revealed the effect of conformers on device performance. These effects are due to the alteration of energy transfer pathways.^53,82,94–97^ Conformers have also been shown to change transition dipole orientation, further potentially affecting exciton delocalization.^98^ Certain applications may benefit from different conformers, such as dye-sensitized solar cells in part because of accommodation of different anchoring options on substrates.^53^ Boltzmann distribution calculations have been used to predict the population of conformers existing in solution.^58^ Squaraine dyes with unaltered central squaric moieties have been found most likely to exist in a *trans*,*anti* (*C*_2h_) symmetry state.

Upon both symmetric and asymmetric substitution, *μ* is relatively unaffected by the type of substitution. Because of this, the exciton hopping interaction occurring between dyes in an aggregate should remain unchanged and may be slightly augmented with substituents. For symmetric substitution, most dyes in the *trans*,*anti* conformations exhibit a Δ*d* of ∼0 D. As shown in Fig. 3, dyes in the *trans*,*anti* conformations have *C*_2h_ symmetry, which, along with the donor–acceptor–donor electronic pattern of squaraine, results in small changes in Δ*d*. In contrast, dyes in the *cis*,*syn* conformations are characterized by *C*_2v_ symmetry and exhibit a non-zero Δ*d*. By only including a single substituent on the dye (*i.e.* asymmetric substitution), the structural symmetry is distorted. Furthermore, substituents with non-zero *σ*_p_ would increase Δ*d*. Specifically for the subsituents studied in this work, the electron withdrawing substituent NO_2_ increases Δ*d* the most. Our computational results suggest that *σ*_p_ can guide the selection of dye candidates with desired electronic and photophysical properties.

Based on the results of this study, the substitution of the squaraine indolenine rings can enhance the dye's excitonic properties. Upon substitution, *μ* is slightly increased for most dyes, indicating that the excitonic hopping interaction between dyes in an aggregate should be enhanced rather than diminished. Similarly, substituents can promote an increase in Δ*d,* which could improve the exciton-exciton interaction energy of the squaraine aggregate.

## 5. Conclusion

Squaraine dyes with varied substituents were investigated to compare their solvation free energy Δ*G*_solv_, static dipole difference Δ*d*, transition dipole moment *μ*, and absorption wavelength *λ*_max_ using DFT and TD-DFT. Changes in these values upon substitution were compared to the empirically derived Hammett constant *σ*_p_ and experimental absorption profiles for the unsubstituted squaraine dye. It was found that the magnitude of *σ*_p_ correlated with Δ*G*_solv_, *μ*, Δ*d*,and *λ*_max_. Δ*G*_solv_ becomes more negative with a larger *σ*_p_ value in water and in solvents similar to a DNA environment. *μ* increases with *σ*_p_ for symmetric substitution patterns. Δ*d* increases with asymmetric substitution and *σ*_p_. *λ*_max_ also increases with *σ*_p_. These findings on the electronic, photophysical, and hydrophobic properties of squaraine dyes can guide the selection of substituted dyes. The ability to control dye properties, when coupled with DNA scaffolding, may make it possible to tailor the performance of dye aggregate materials for excitonic systems and applications.

## Author contributions

German Barcenas: investigation (lead), writing – original draft (lead), methodology (equal), formal analysis (lead), data curation (lead). Austin Biaggne: investigation (supporting), writing – original draft (supporting), methodology (equal), formal analysis (supporting), data curation (supporting). Olga A. Mass: conceptualization (equal), validation (lead), writing – review and editing (equal), supervision (supporting). Christopher K. Wilson: validation (supporting). Olena M. Obukhova: resources (supporting). Olga S. Kolosova: resources (supporting). Anatoliy L. Tatarets: resources (supporting). Ewald Terpetschnig: resources (supporting), writing – review and editing (equal). Ryan D. Pensack: conceptualization (equal), writing – review and editing (equal). Jeunghoon Lee: conceptualization (equal), writing – review and editing (equal). William B. Knowlton: conceptualization (equal), project administration (supporting), writing – review and editing (equal). Bernard Yurke: conceptualization (equal), writing – review and editing (equal), supervision (supporting). Lan Li: conceptualization (equal), project administration (lead), writing – review and editing (equal), supervision (lead).

## Conflicts of interest

There are no conflicts to declare.

## Supplementary Material
